# Understanding the molecular mechanisms of macrophage polarization and metabolic reprogramming in endometriosis: A narrative review

**DOI:** 10.1002/rmb2.12488

**Published:** 2022-10-17

**Authors:** Hiroshi Kobayashi, Shogo Imanaka

**Affiliations:** ^1^ Department of Gynecology Ms.Clinic MayOne Kashihara, Nara Japan; ^2^ Department of Obstetrics and Gynecology Nara Medical University Kashihara, Nara Japan

**Keywords:** endometriosis, macrophages, metabolic reprogramming, phenotype

## Abstract

**Background:**

Endometriosis is an estrogen‐dependent disease and causes pelvic pain and infertility. The limits of current pharmacotherapy in women who desire to become pregnant prompt the development of various targeted molecules for more effective treatment. A review article focused on the unique aspect of cellular metabolic reprogramming of endometriotic cells has been reported. The cellular metabolic pathways are reprogrammed to adapt to a variety of environmental stresses (e.g., nutrient starvation or glucose deprivation, hypoxic stress, excessive reactive oxygen species generation, and other environmental factors). This review aims to summarize macrophage polarization and metabolic reprogramming in endometriosis.

**Methods:**

A literature search was performed between January 2000 and March 2022 in the PubMed and Google Scholar databases using a combination of specific terms.

**Results:**

Macrophage cellular metabolism has a marked influence on its phenotype and function. Preclinical studies showed that metabolic conversion toward glycolysis or oxidative phosphorylation drives macrophage polarization to M1 or M2 phenotype, respectively. Such cellular metabolic rewiring can offer new therapeutic opportunities.

**Conclusion:**

A better understanding of metabolic reprogramming biology in endometriosis‐associated macrophages is essential in considering novel therapeutic approach for endometriosis. However, there are currently no detailed studies on therapeutic strategies targeting the cellular metabolic properties of endometriosis‐associated macrophages.

## 1 INTRODUCTION

Endometriosis is an estrogen‐dependent condition that causes pelvic pain and infertility.^1^ Endometriosis is characterized by inflammation, angiogenesis, tissue remodeling, and immune evasion.^1^ The growth of endometriosis is determined by its interactions with various immune cells, including macrophages, natural killer (NK) cells, and T regulatory cells (Tregs).^2^, ^3^ Macrophages are abundant immune cells found in peritoneal fluid and also present within the lesions, and can either kill endometriotic cells or often foster lesion growth.^4^ Moreover, macrophages are critical for inflammation and innervation, a pain‐promoting role.^5^ Therefore, increased numbers of immune cells and their dysfunction play a key role in the pathogenesis of endometriosis.

In addition, like cancer, endometriosis requires the fuel to adapt and grow in hypoxic and harsh environments.^6^, ^7^ Several molecular similarities between cancer and endometriosis have been reported in terms of the reprogramming of energy metabolism.^8^, ^9^, ^10^ Human cell utilizes glucose as a primary source of energy, and adenosine triphosphate (ATP) is generated from glucose via glycolysis and mitochondrial oxidative phosphorylation. Endometriotic cells acquire unique cellular metabolic features that enable them to meet their energy demands and survive even under unfavorable environment. Recent studies revealed that cancer cells and endometriotic cells preferentially use glycolysis over glucose oxidation for energy generation.^6^, ^11^, ^12^, ^13^ A review article focusing on the unique aspect of metabolic reprogramming of endometriosis has been reported.^7^ The cellular metabolic shift from oxidative phosphorylation to glycolysis suppresses endometriotic cell death through decreasing the level of mitochondrial reactive oxygen species (ROS) generation. Changing the specific metabolic switch toward oxidative phosphorylation leads to cell death by causing increased ROS production and severe oxidative stress. These findings open up new avenues for the development of optimal strategies for nonhormonal treatment of endometriosis.^6^

In addition, macrophages are located near endometriotic cells and contribute to the homeostatic regulation within the immune microenvironment of endometriosis. Macrophage polarization contributes to the control of ectopic endometrial cell initiation and progression. M1 and M2 macrophages have been suggested to inhibit and promote the development of endometriosis, respectively. In the field of oncology, M1 and M2 macrophages are thought to be predominantly dependent on glycolysis and oxidative phosphorylation, respectively.^10^, ^14^ However, it has not been understood how metabolic reprogramming in endometriosis affects the immune response of macrophages. Understanding the interaction between endometriosis and macrophages, in addition to metabolic remodeling in endometriotic cells, may elucidate the pathogenesis of endometriosis and offer novel therapeutic approaches. The review aims to discuss the crosstalk between endometriotic cells and macrophages and summarize the current status on the molecular mechanisms of metabolic reprogramming and macrophage polarization.

## 2 METHODS

### 2.1 Search strategy and selection criteria

**FIGURE 1:**
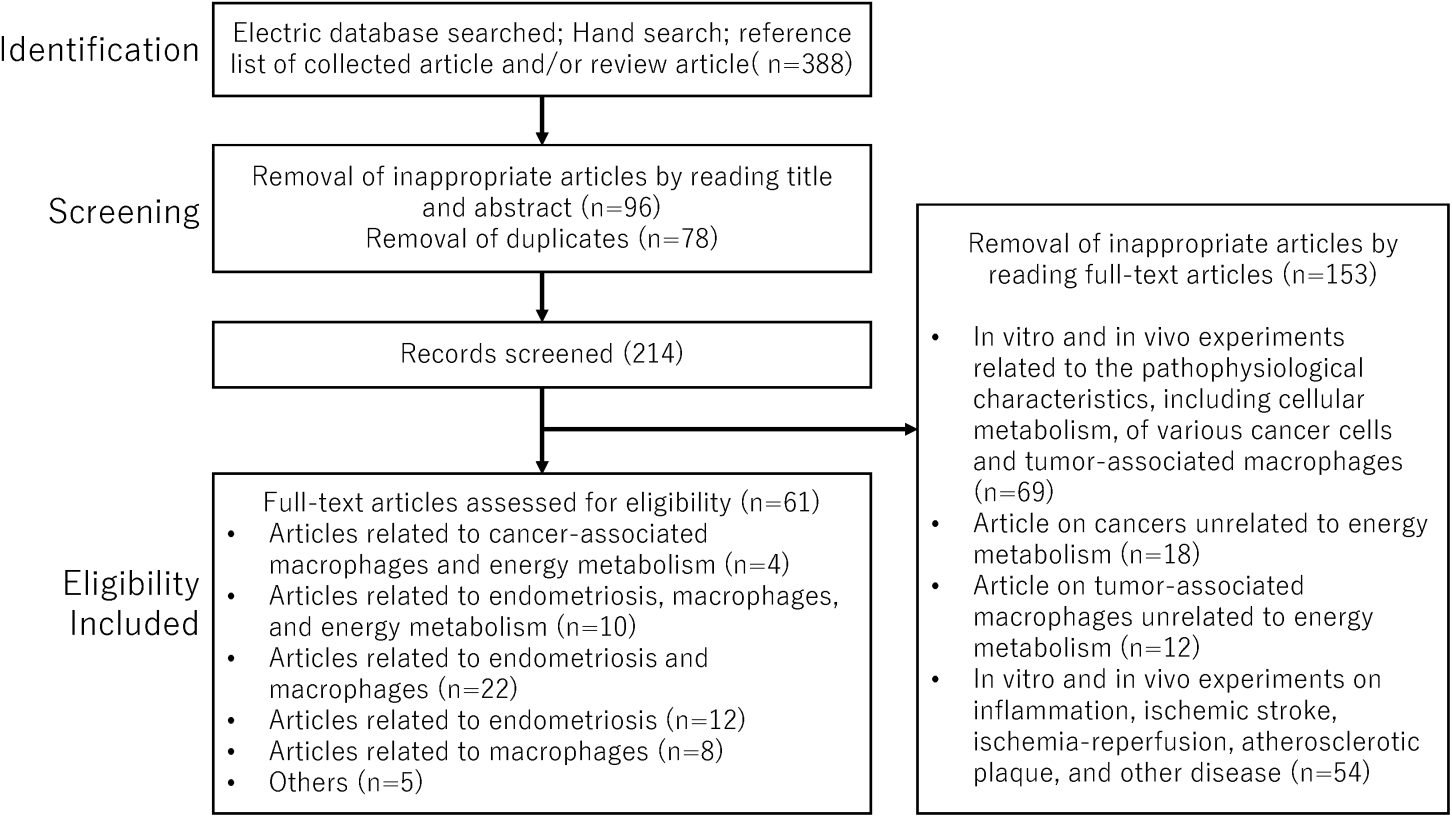
The number of articles identified by searching for keyword combinations. This figure shows the number of articles identified by keyword combinations and the number of records identified through database searching, records after duplicate removal, records screened, removal of inappropriate articles by reading full‐text articles, and full‐text articles assessed for eligibility.

A computerized literature search was performed to identify relevant studies. The study was conducted in accordance with the PRISMA (Preferred Reporting Items for Systematic Reviews and Meta‐Analyses) guidelines updated in 2020^15^ (see Table S1). PubMed and Google Scholar electronic databases published between January 2000 and December 2021 were searched, combining the following keywords: *Endometriosis*, *Macrophages*, *Metabolic shift*, *Metabolic reprogramming*, *Polarization*, *Phenotype*, and *Treatment*. The inclusion criteria were publications of original studies and review papers and reference lists of the included studies. The exclusion criteria were duplicates, studies in languages other than English, letters to the editor, poster presentation, and literature unrelated to the research topic. The literature search was conducted using keywords with the following search combination: (*Endometriosis* OR *Macrophages*) AND (*Metabolic shift* OR *Metabolic reprogramming*) AND (*Polarization* OR *Phenotype*) AND *Treatment*. After searching, we excluded publications that contained the keyword “*Tumor*”. The first identification phase includes records identified through a database search (Figure 1). Identified titles and abstracts were screened in the first stage. Duplicates were removed during the second screening phase, and titles, abstracts, and full‐text articles were read to remove inappropriate papers. Citation tracking was manually conducted to identify additional relevant articles. The final eligibility phase included the full‐text articles for analysis after excluding those for which detailed data cannot be extracted. Two authors (HK and SI) independently assessed the identified articles for eligibility, inclusion, and exclusion and subsequently full‐text articles. Initial disagreements were resolved by consensus. As shown in Figure 1, a systematic search resulted in a final selection of 61 articles, excluding articles containing the keyword “Tumor”. These articles include articles related to cancer‐associated macrophages and energy metabolism (*n* = 4), those related to endometriosis, macrophages, and energy metabolism (*n* = 10), those related to endometriosis and macrophages (*n* = 22), those related to endometriosis (*n* = 12), those related to macrophages (*n* = 10), and others (*n* = 3). The last computerized literature search was conducted on 25 January 2022.

## 3 RESULTS

### 3.1 Selection of studies

The search in the PubMed and Google Scholar electronic databases provided 388 literature citations (Figure 1). Following the removal of overlaps, 214 records were obtained, of which 153 were excluded, and 61 met the inclusion and exclusion criteria.

### 3.2 Crosstalk between endometriotic cells and diverse cell types

**FIGURE 2:**
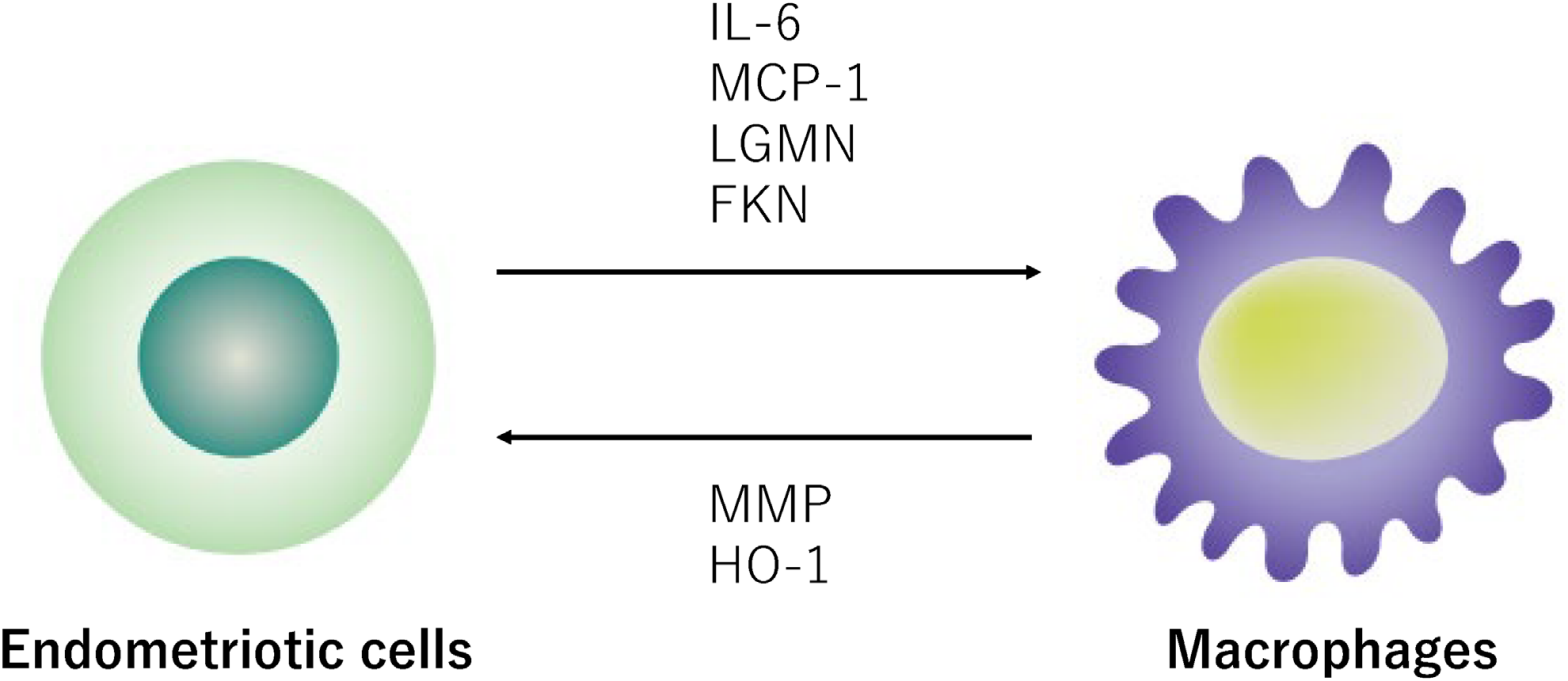
Crosstalk and interaction between endometriotic cells and macrophages. Mediators (e.g., IL‐6, MCP‐1, LGMN, and FKN) produced by human endometriotic cells modulate the activation state of macrophages, which in turn promotes the progression of endometriosis. Macrophages co‐cultured with endometriotic cells in the hypoxic milieu upregulate HO‐1 and MMP expression and promote survival of endometriotic cells.FKN, fractalkine; HO‐1, heme oxygenase‐1; IL‐6, interleukin‐6; LGMN, legumain; MCP‐1, monocyte chemoattractant protein‐1; MMP, matrix metalloproteinase.

The proportion of immune cells, including macrophages, NK cells, and CD4^+^Foxp3^+^ regulatory T cells (Tregs), is increased in the peritoneal fluid of women with endometriosis.^2^, ^3^, ^16^ Peritoneum and immune cells play active roles in the pathogenesis of endometriosis and are key components of the microenvironment with diverse functions, including the crosstalk between endometriotic cells and the surrounding cells. Here, this section focuses on the crosstalk and interaction between endometriotic cells and macrophages (Figure 2).

In general, macrophages have dual functions in tissue injury and repair. Although macrophages exhibit a phenotype that varies with disease stage,^17^ infiltrating macrophages in endometriosis lesions often play a role in tissue remodeling and impaired phagocytic ability.^2^ Furthermore, endometriotic stromal cells co‐cultured with macrophages have been reported to increase the survival and invasive ability.^4^ For example, reciprocal signaling between endometriotic cells and macrophages may promote disease progression through the secretion and expression of several molecular mediators (e.g., tumor necrosis factor‐α (TNF‐α), interleukin‐6 (IL‐6)), monocyte chemoattractant protein‐1 (MCP‐1), legumain (LGMN), fractalkine (FKN), and transforming growth factor‐β (TGF‐β) from endometriotic cells.^18^, ^19^, ^20^, ^21^ Endometriotic cells treated with TNF‐α activate peritoneal macrophages via pro‐inflammatory cytokines such as IL‐6 and MCP‐1, which has a vital role in initiating, progressing, and protecting endometriosis through the promotion of invasion and proliferation of endometriotic cells.^18^ LGMN is a cysteine protease that is involved in the processing of endogenous proteins for major histocompatibility complex class II presentation and promotes cell migration, invasion, angiogenesis, and proliferation.^19^ FKN, a chemokine involved in the adhesion and migration of immune cells, alters cytokine production with upregulation of IL‐10 and downregulation of IL‐12, implicating a protective effect of macrophage against endometriosis progression.^20^ Endometriotic cell‐derived TGF‐β1 has a protective effect on oxidative tissue injury via the upregulation of heme oxygenase‐1 (an antioxidant enzyme) in macrophages.^21^ Therefore, the crosstalk between endometriotic cells and macrophages may contribute to the pathogenesis and progression of endometriosis.^21^ As described later, these mediators are important in skewing the M1/M2 balance toward an anti‐inflammatory profile and protecting endometriotic cells from immune attack.

### 3.3 Macrophage phenotype in endometriosis

**FIGURE 3:**
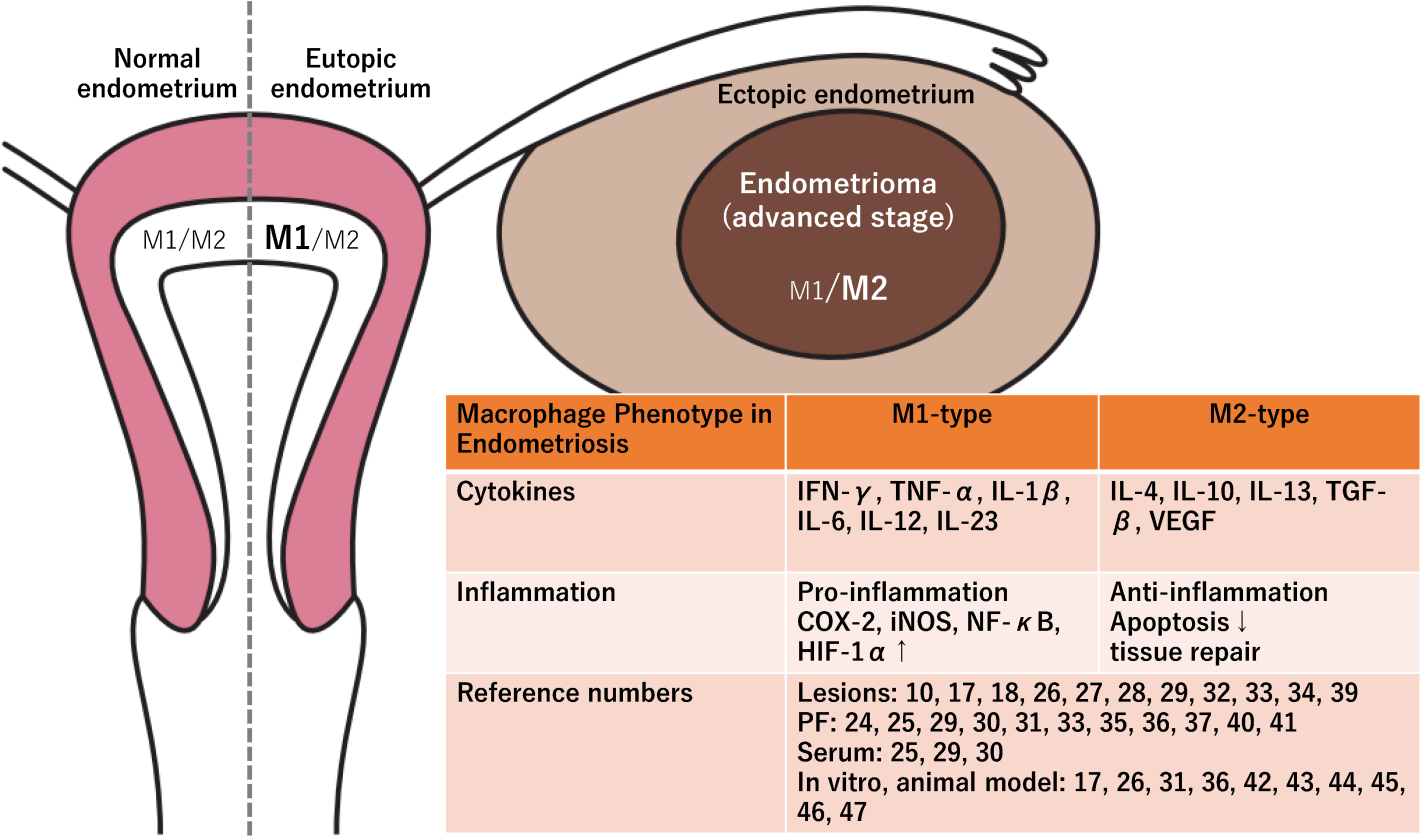
Phenotypic and functional features of macrophages in normal endometrium, eutopic endometrium, and ectopic endometrium. The brown table shows pro−/anti‐inflammatory cytokines and chemokines that are differentially expressed in serum, ascitic fluid, animal models, and ectopic lesions in women with endometriosis. COX‐2, cyclooxygenase 2; HIF‐1α, hypoxia‐inducible factor‐1alpha; IFN‐γ, interferon‐gamma; IL‐1β, interleukin‐1beta; IL‐4, interleukin‐4; IL‐6, interleukin‐6; IL‐10, interleukin‐10; IL‐12, interleukin‐12; IL‐13, interleukin‐13; IL‐23, interleukin‐23; iNOS, inducible nitric oxide synthase; NF‐κB, nuclear factor kappa B; TGF‐β, transforming growth factor‐beta; TNF‐α, tumor necrosis factor‐alpha; VEGF, vascular endothelial growth factor; and PF, peritoneal fluid.

Endometriosis is characterized by progressive inflammation and immune evasion. Among diverse immune cells, macrophages play key roles in inflammation, tissue injury, repair, and regeneration in the endometriosis milieu.^22^ Unlike the physiological wound healing, excessive inflammation and repair in endometriosis lesions lead to the deposition of extracellular matrix, fibrosis, and tissue dysfunction via persistent activation of macrophages. As a result, tissue integrity and physiological function cannot be restored.^5^, ^22^ As shown in Figure 3, with a few exceptions,^23^, ^24^ studies showed that levels of typical Th2 cytokines (e.g., IL‐4, IL10, IL‐13, or TGF‐β) in serum, peritoneal fluid, and ectopic lesions of patients with endometriosis were higher than those of normal patients, whereas levels of typical Th1 cytokines (e.g., interferon‐γ [IFN‐γ] or IL‐12) were suppressed.^18^, ^25^, ^26^, ^27^, ^28^, ^29^, ^30^, ^31^, ^32^, ^33^, ^34^, ^35^, ^36^, ^37^ Indeed, in vivo experimental animal models revealed that pharmacological depletion of peritoneal macrophage suppressed peritoneal fluid MCP‐1 levels, thereby attenuating the initiation and growth of endometriosis implants.^38^

In addition, infiltrating macrophages exhibit a dynamic change in functionally unique phenotype that varies with disease stage or severity.^17^ Macrophages are classified into a pro‐inflammatory type (the classically activated M1 type) and an anti‐inflammatory/pro‐resolving type (the alternatively activated M2 type) according to their role.^9^, ^10^ M1 macrophages are characterized by their ability to cause inflammation and kill pathogens by producing cyclooxygenase 2 and inducible nitric oxide synthase (iNOS) through upregulating diverse pro‐inflammatory cytokines, including TNF‐α, IL1‐β, IL‐6, IL‐12, and IL‐23.^10^ For example, NF‐κB signaling pathway is involved in the phenotypic conversion of M2 macrophages to M1 macrophages.^10^ M1 macrophages gradually decrease during endometriosis stage progression.^10^ In contrast, M2 macrophages display an anti‐inflammatory, immunosuppressive, angiogenesis, neuroangiogenesis, and tissue repair character, by producing IL‐4, IL‐10, IL‐13, IL‐17A, TGF‐β, vascular endothelial growth factor A, endothelial growth factor, and platelet‐derived growth factor.^10^, ^39^, ^40^ For example, TGF‐β switches macrophages from M1 to M2 through activating the small mothers against decapentaplegic pathway.^26^ Previous studies have indicated that the M2 phenotype becomes predominant in the peritoneal environment of women with endometriosis^41^ or in an endometriosis animal model^42^, ^43^, ^44^ and that a switch from the M1‐ toward a M2‐polarized phenotype mediates the process of immunosuppression, a hallmark of disease progression.^17^, ^26^ M2 macrophages have positive effects on multiple steps in endometriosis growth.^39^ Therefore, M1 and M2 macrophages may be involved in suppressing or promoting the development of endometriosis, respectively.^39^

Furthermore, the characteristic and phenotype of macrophages are dependent on its local microenvironment.^42^, ^43^ In an experimental model in which human endometrial tissue was grafted into immunodeficient mice, macrophage phenotype was altered from M1 to M2 over time.^17^ Moreover, macrophage polarization depends on the specific site and the microenvironment generated by the lesion, possibly with resident macrophages being M1 subtype and peritoneal macrophages being M2 subtype.^5^ It has been reported that M1 macrophages are enriched in the eutopic endometrium, whereas the macrophages of the ectopic endometrium are polarized toward an M2‐type.^18^ M1/M2 macrophage polarization is regulated by several genes (i.e., spleen tyrosine kinase, bridging integrator 2, metalloproteinase 12, chemokine receptor 5, macrophage mannose receptor 1, and TNF receptor type 1‐associated death domain) that are differentially expressed in endometriosis.^45^, ^46^ These results suggest that macrophages can be activated and polarized by the endometriosis‐derived soluble factors.^47^

### 3.4 Cellular metabolism in endometriosis

#### 3.4.1 Overview of glucose metabolism

Cell proliferation is influenced by energy metabolism, including aerobic and anaerobic glycolysis. Endometriotic cells convert significant amounts of glucose into energy to undergo growth in hypoxic milieus.^7^ Recent studies suggest that reprogramming of energy metabolism is a hallmark of endometriosis development.^6^, ^11^, ^12^, ^13^ First, we focus on how endometriotic cells dynamically reconstruct signaling pathways that regulate glucose metabolism in the maintenance of energy homeostasis and summarize the underlying key regulators. Second, we discuss how changes in energy metabolism allow macrophages to fine‐tune polarization.

**FIGURE 4:**
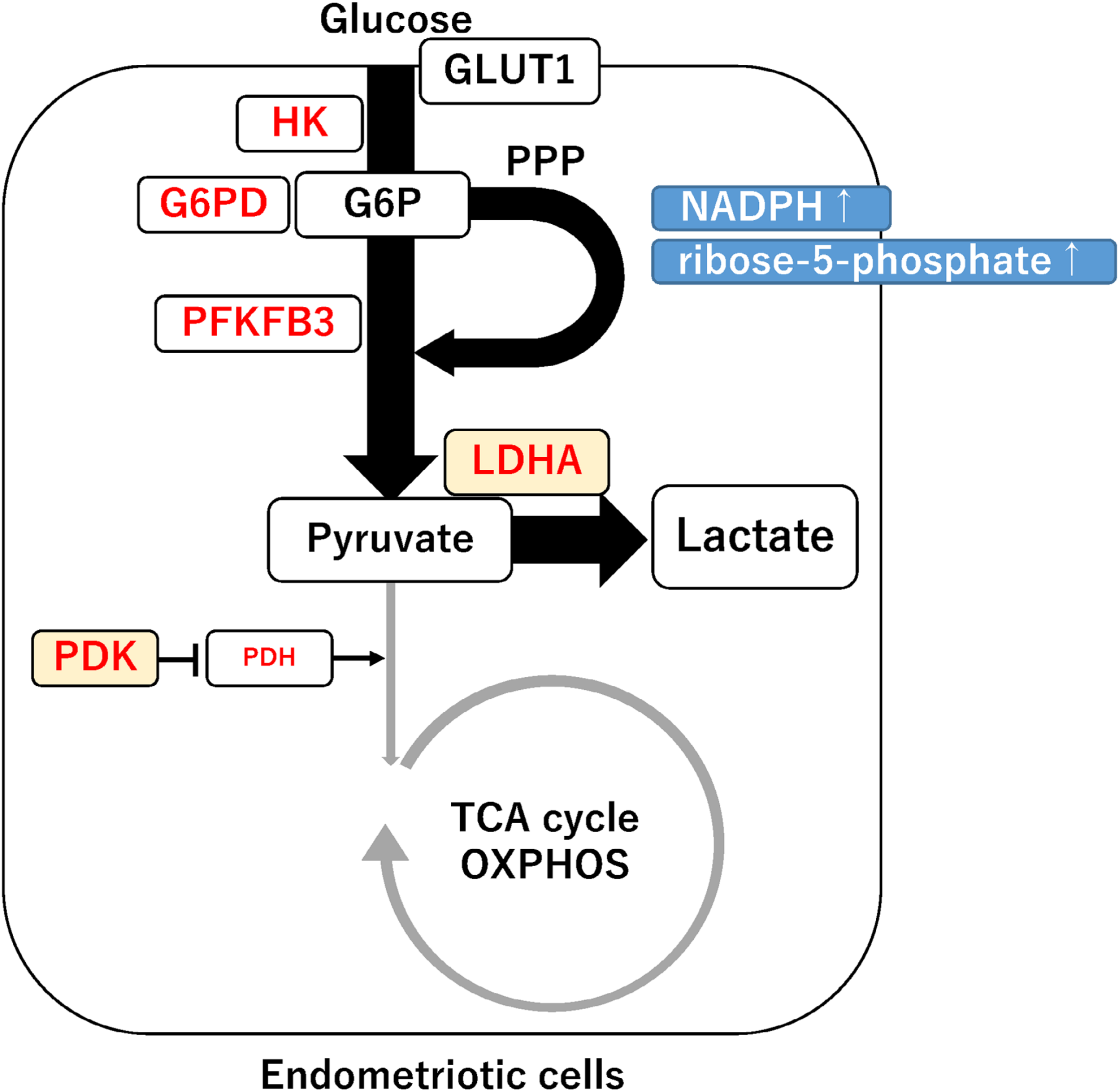
Metabolic reprogramming in endometriosis. Red letters indicate glycolytic enzymes, and PDK and LDHA play a critical role in metabolic reprogramming from oxidative phosphorylation to glycolysis. G6P, glucose‐6‐phosphate; GLUT, glucose transporter; PFKFB3, 6‐phosphofructo‐2‐kinase/fructose‐2,6‐biphosphatase 3; OXPHOS, oxidative phosphorylation; PDH, pyruvate dehydrogenase; PDK, pyruvate dehydrogenase kinase; PPP, pentose phosphate pathway; and TCA, tricarboxylic acid.

We initially overview the metabolic networks that supply energy to human cell (Figure 4). Human cell is able to produce ATP via the major metabolic pathways, including glycolysis and pentose phosphate pathway (PPP) in the cytoplasm as well as tricarboxylic acid (TCA) cycle and oxidative phosphorylation within the mitochondria.^10^ One glucose molecule produces two ATP in the glycolytic metabolic pathway, while 32–38 molecules of ATP are generated through the TCA cycle and oxidative phosphorylation.^10^ The PPP branching from glycolysis produces nicotinamide adenine dinucleotide phosphate (NADPH), a regulator of the antioxidant system, and ribose‐5‐phosphate, a precursor of nucleotides.^10^ Thus, aerobic glycolysis is an inefficient ATP production pathway, but confers survival benefits through the synthesis of ribose, amino acids, fatty acids, nucleotides, and antioxidants.^10^ Hexokinase 2 (HK2), glucose‐6‐phosphate dehydrogenase, lactate dehydrogenase A (LDHA), pyruvate dehydrogenase kinase (PDK), and pyruvate dehydrogenase (PDH) are key enzymes that play an important role in regulating the cellular energetic pathways.^48^ Among these enzymes, the PDK‐PDH axis is believed to play a central role as a cellular metabolic switch.^48^ The PDH complex converts pyruvate to acetyl‐CoA, which activates the transfer of acetyl‐CoA to the TCA cycle.^48^ In contrast, PDK inhibits the activation of the PDH complex.^48^ Since PDK activity is suppressed in normal cells, the PDH complex activates the production of ATP through the TCA cycle. Changing environmental conditions are reported to accelerate a cellular metabolic switch from glycolysis to mitochondrial oxidative phosphorylation or vice versa.^7^

#### 3.4.2 Metabolic reprogramming in endometriosis

More detailed information on the cellular metabolic profiles in endometriosis can be found in ref. [7]. Ectopic endometrial tissue specimens and adjacent peritoneal lesions were obtained from patients with endometriosis who underwent surgery. Endometriotic stromal cells and peritoneal cells derived from isolated ectopic endometriotic lesions were cultured and used for further cellular metabolism experiments.^11^, ^12^, ^13^ Endometriotic cells are thought to be able to survive in harsh environments by altering their cellular metabolism from oxidative phosphorylation to glycolysis through upregulating the glucose transporter‐1 (GLUT1) and glycolytic enzymes^6^, ^11^, ^12^, ^13^, ^49^, ^50^, ^51^, ^52^ (Figure 4). In response to changes in environmental conditions, endometriotic cells alter the expression profile and pathways of cellular metabolic enzymes. The cellular metabolic pathways of endometriosis closely resemble the metabolic activity in cancer, the so‐called “Warburg effect”, involving the increased utilization of glycolysis rather than mitochondrial oxidative phosphorylation.^6^ As shown in Figure 3, there are many reports on the expression of cytokines, growth factors, and angiogenic factors in the sera, peritoneal fluid, and endometriotic tissue (e.g., peritoneal lesion, ovarian endometriomas, and deep infiltrating endometriosis). However, eutopic and ectopic endometrial stromal cells as well as peritoneal cells collected from patients with endometriosis were mainly used for in vitro cellular metabolism experiments. Therefore, whether there are differences in the cellular metabolism between different types of endometriosis remains unclear.

### 3.5 Macrophage polarization based on metabolic reprogramming

**FIGURE 5:**
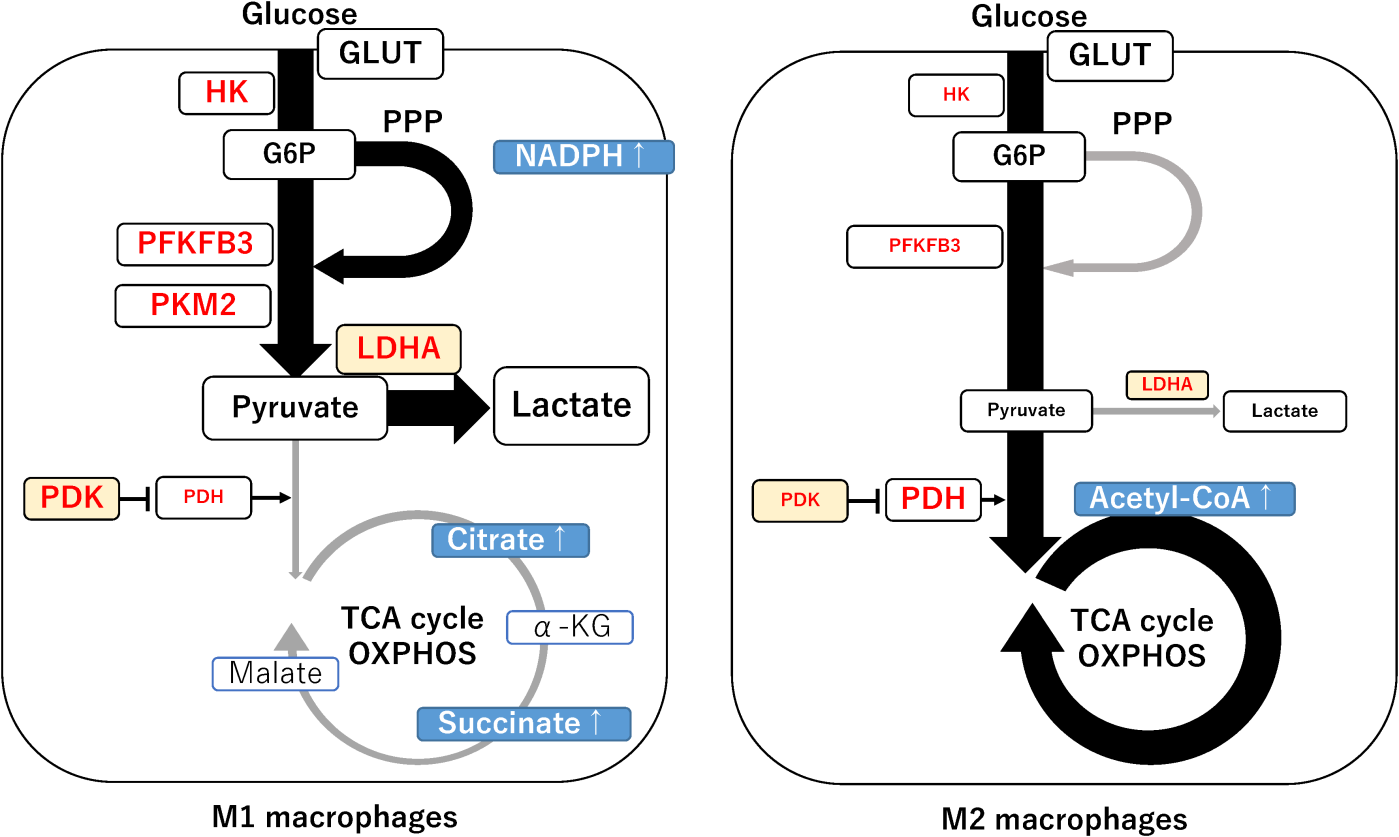
Macrophage polarization based on metabolic reprogramming. This figure depicts general cellular metabolic profiles of tumor‐associated macrophages because of the small number of experimental models that involve the cellular metabolic rewiring in endometriosis‐associated macrophages. Left figure: M1 macrophages upregulate the glycolysis enzymes (e.g., GLUT1, PFKFB3, PKM2, LDHA, and PDK), producing increased glycolytic flux. The oxidative PPP activity is crucial for M1 macrophages for anti‐oxidant defense mechanisms and fatty acid biosynthesis.^56^ Interruption of TCA cycle results in accumulation of citrate and succinate, leading to HIF1α stabilization and activation of glycolytic enzyme genes. Right figure: In contrast, M2 macrophages are characterized by a cellular metabolic switch toward oxidative phosphorylation for the functions involved in tissue repair.

Next, we discuss how metabolic reprogramming is involved in macrophage polarization (Figure 5). Table 1 summarizes cellular metabolic pathways and enzymes activated in M1 and M2 macrophages. The M1/M2 shift in macrophage phenotypes has been reported to be closely associated with the progression of endometriosis^17^; however, research on metabolic reprogramming in endometriosis‐related macrophages is still in its infancy. The following data on metabolic reprogramming in macrophage polarization were primarily obtained from the field of molecular biology and oncology. Cellular metabolic shifting between glycolysis and mitochondrial oxidative phosphorylation is known to be implicated in the phenotypic and functional changes of macrophages, especially tumor‐associated macrophages.^10^, ^53^ M1 and M2 macrophages rely primarily on glycolysis and mitochondrial oxidative phosphorylation, respectively, to generate the energy.^10^, ^14^ M1 macrophages exhibit increased glycolysis along with increased metabolic reprogramming toward aerobic glycolysis, PPP, and fatty acid synthesis (Figure 5, left). The glycolytic flux is controlled by multiple steps, such as glucose transporters (e.g., GLUT1), oxidative pathways (e.g., PPP and fatty acid synthesis), and glycolytic enzymes (e.g., PFKFB, pyruvate kinase M2 [PKM2], LDHA, PDK, and PDH), signal metabolites (e.g., citrate, succinate, and itaconate), and transcription factors (e.g., TGF‐β and HIF‐1α).^10^, ^14^, ^48^, ^54^, ^55^, ^56^, ^57^, ^58^ In particular, the upregulation of GLUT1, PFKFB3, PKM2, and PDK1 expression facilitates a rapid glucose uptake and an accelerated glycolytic flux.^54^, ^55^ The PPP provides NADPH oxidase to produce ROS for killing pathogens or for physiological processes, including cell proliferation and differentiation.^56^ Additionally, the upregulation of LDHA and PDK1 expression promotes the conversion of pyruvate into lactate, thus limiting pyruvate to acetyl‐CoA conversion in mitochondria.^10^, ^48^, ^58^ This may be accompanied by mitochondrial dysfunctions. Disrupted TCA cycle results in the accumulation of signal metabolites (e.g., citrate, succinate, and itaconate).^10^ For example, succinate impairs prolyl hydroxylase (PHD) activity and stabilizes HIF‐1α, that leads to metabolic reprogramming toward glycolysis.^10^ Collectively, in M1 macrophages, cellular metabolism is shifted from mitochondrial oxidative phosphorylation toward glycolysis, which in turn increases glucose uptake and lactate production, amplifying the innate defense mechanisms and immune surveillance.

**TABLE 1:** Metabolic pathways and enzymes activated in M1 and M2 macrophages

Macrophage phenotype	M1‐type	M2‐type
Metabolic sift	Toward glycolysis	Toward oxidative phosphorylation (glucose oxidation)
Activated metabolic pathways	PPP Fatty acid synthesis Ribose synthesis NADPH synthesis	Fatty acid oxidation Glutaminolysis
Activated metabolic enzymes	GLUT1, PDK, LDHA	PDH

In contrast, in M2 macrophages, oxidative phosphorylation is the major metabolic pathway, and pyruvate is transported to mitochondria and converted to acetyl‐CoA by PDH^10^ (Figure 5, right). Metabolic conversion from glycolysis to oxidative phosphorylation increases IL‐10 production via AMPK‐dependent activation of p38 mitogen‐activated protein kinase (p38 MAPK), c‐Jun N‐terminal kinase (c‐JUK), and cyclic AMP‐responsive element‐binding protein.^59^ The metabolic shift toward oxidative phosphorylation promotes the polarization of macrophages to the M2 phenotype, which shows unique functions, such as cell proliferation, tissue repair and wound healing, and fibrosis.^14^ Interestingly, recent experimental animal models of endometriosis showed that the elevated M1−/M2‐phenotype ratio was significantly reduced by increased mitochondrial biosynthesis,^60^ indicating that changes in metabolic pathways can regulate macrophage polarization. This suggests that the metabolic shift from glycolysis toward oxidative phosphorylation can convert macrophage phenotype from M1 to M2. However, the dynamic spatiotemporal regulation process and mechanisms underlying the metabolic switch are not yet fully understood in endometriosis.

Collectively, metabolic reprogramming is accompanied by changes in macrophage polarization. These results were mainly obtained from tumor‐infiltrating macrophages. Unfortunately, metabolic reprogramming in endometriosis‐associated macrophages has not been investigated in detail.

## 4 DISCUSSION

This review summarized the crosstalk and cellular metabolic regulation in endometriotic cells and macrophages, and the factors and pathways relevant to metabolic reprogramming.

The crosstalk between endometriotic cells and immune cells induces altered immune response to facilitate immune evasion, which contributes to endometriotic cell growth and progression. Endometriosis‐associated immune cells, including macrophages and Treg, are a heterogeneous population that plays diverse functions in immune responses. In the field of oncology, metabolic conversion determines the phenotype of macrophages, supporting that cellular metabolism regulates the immune responses.^10^, ^14^, ^17^, ^53^ We mainly summarized the cellular metabolic profile of tumor‐associated macrophages because of the small number of experimental models that involve the cellular metabolic rewiring in endometriosis‐associated macrophages.^60^ Macrophages and cancer cells require metabolic reprogramming to maximize fitness under inflammatory environments. For example, metabolic conversion toward glycolysis or glucose oxidation drives macrophage polarization to M1 or M2 phenotype, respectively.^61^ M1 macrophages, cancer cells, and endometriotic cells can shift their metabolic pathway toward glycolysis, while M2 macrophages shift their cellular metabolism toward oxidative phosphorylation.^61^ These studies suggest cellular metabolic similarities between cancer, endometriosis, and M1 macrophages.^6^, ^7^, ^11^, ^12^, ^13^ However, it is currently unclear whether macrophages directly affect the cellular metabolic properties in endometriosis lesions and vice versa. Further investigation is required for the validation of cellular metabolic pathways involved in the polarization of macrophage associated with endometriosis.

In conclusion, this review summarizes the pathophysiology of endometriosis in terms of metabolic reprogramming and macrophage polarization and provides new perspectives and opportunities for understanding the cellular metabolic biology. Studies on the dynamic spatiotemporal regulation of cellular metabolic pathways of endometriotic cells and macrophages have just begun.

## AUTHOR CONTRIBUTIONS

Conception and design: HK. Acquisition of data: HK and SI. Analysis and interpretation of data: HK and SI. Writing the manuscript: HK. The final version of the manuscript has been read and approved by all authors.

## CONFLICT OF INTEREST

The authors (HK and SI) have no conflicts of interest to declare.

## Supporting information


Table S1.

## Data Availability

The datasets generated during the present study are available from Hiroshi Kobayashi.
